# A phase II randomized trial of metastasis-directed therapy with alpha emitter radium-223 in men with oligometastatic castration-resistant prostate cancer (MEDAL)

**DOI:** 10.1186/s12894-023-01202-z

**Published:** 2023-03-06

**Authors:** Soichiro Yoshida, Taro Takahara, Yuki Arita, Masaya Ito, Sara Hayakawa, Tomohiko Oguchi, Yoshinobu Komai, Noboru Numao, Takeshi Yuasa, Masaharu Inoue, Hiroki Ushijima, Shigehiro Kudo, Yasumasa Shimano, Yuki Nakamura, Yusuke Uchida, Sho Uehara, Hajime Tanaka, Hiroshi Yaegashi, Kouji Izumi, Minato Yokoyama, Yoh Matsuoka, Yasuo Yoshioka, Koji Konishi, Katsuyuki Nakanishi, Akira Nagahara, Akihiro Hirakawa, Ryuji Koike, Fumitaka Koga, Kazuo Nishimura, Atsushi Mizokami, Junji Yonese, Yukio Kageyama, Ryoichi Yoshimura, Yasuhisa Fujii

**Affiliations:** 1 Department of Urology, Tokyo Medical and Dental University, 1-5-45 Yushima, Bunkyo-Ku, Tokyo, 113–8510 Japan; 2 Department of Biomedical Engineering, Tokai University School of Engineering, Kanagawa, Japan; 3 Department of Radiology, Advanced Imaging Center, Yaesu Clinic, Tokyo, Japan; 4 Department of Radiology, Keio University School of Medicine, Tokyo, Japan; 5 Department of Urology, Tokyo Metropolitan Cancer and Infectious Diseases Center Komagome Hospital, Tokyo, Japan; 6 Department of Radiation Oncology, Department of Radiology, Tokyo Metropolitan Cancer and Infectious Diseases Center Komagome Hospital, Tokyo, Japan; 7 Department of Genitourinary Oncology, Cancer Institute Hospital of Japanese Foundation for Cancer Research, Tokyo, Japan; 8 Department of Urology, Saitama Cancer Center, Saitama, Japan; 9 Department of Radiation Oncology, Saitama Cancer Center, Saitama, Japan; 10 Department of Integrative Cancer Therapy and Urology, Kanazawa University Graduate School of Medical Science, Ishikawa, Japan; 11 Radiation Oncology Department, Cancer Institute Hospital of Japanese Foundation for Cancer Research, Tokyo, Japan; 12 Department of Radiation Oncology, Osaka International Cancer Institute, Osaka, Japan; 13 Department of Diagnostic and Interventional Radiology, Osaka International Cancer Institute, Osaka, Japan; 14 Department of Urology, Osaka International Cancer Institute, Osaka, Japan; 15 Department of Clinical Biostatistics, Graduate School of Medical and Dental Sciences, Tokyo Medical and Dental University, Tokyo, Japan; 16 Department of Medical Innovation Promotion Center, Tokyo Medical and Dental University, Tokyo, Japan; 17 Department of Radiation Therapeutics and Oncology, Tokyo Medical and Dental University, Tokyo, Japan

**Keywords:** Prostatic Neoplasms, Castration-resistant, Radium-223, Radiotherapy, Neoplasm metastasis, Randomized controlled trial

## Abstract

**Background:**

The significance of metastasis-directed therapy for oligometastatic prostate cancer has been widely discussed, and targeted therapy for progressive sites is a feasible option as a multidisciplinary treatment for castration-resistant prostate cancer (CRPC). When oligometastatic CRPC with only bone metastases progresses after targeted therapy, it tends to progress as multiple bone metastases. The progression of oligometastatic CRPC after targeted therapy may be due in part to the presence of micrometastatic lesions that, though undetected on imaging, were present prior to targeted therapy. Thus the systemic treatment of micrometastases in combination with targeted therapy for progressive sites is expected to enhance the therapeutic effect. Radium-223 dichloride (radium-223) is a radiopharmaceutical that selectively binds to sites of increased bone turnover and inhibits the growth of adjacent tumor cells by emitting alpha rays. Therefore, for oligometastatic CRPC with only bone metastases, radium-223 may enhance the therapeutic effect of radiotherapy for active metastases.

**Methods:**

This phase II, randomized trial of *Me*tastasis-*D*irected therapy with *AL*pha emitter radium-223 in men with oligometastatic CRPC (MEDAL) is designed to assess the utility of radium-223 in combination with metastasis-directed radiotherapy in patients with oligometastatic CRPC confined to bone. In this trial, patients with oligometastatic CRPC with three or fewer bone metastases on whole-body MRI with diffusion-weighted MRI (WB-DWI) will be randomized in a 1:1 ratio to receive radiotherapy for active metastases plus radium-223 or radiotherapy for active metastases alone. The prior use of androgen receptor axis-targeted therapy and prostate-specific antigen doubling time will be used as allocation factors. The primary endpoint will be radiological progression-free survival against progression of bone metastases on WB-DWI.

**Discussion:**

This will be the first randomized trial to evaluate the effect of radium-223 in combination with targeted therapy in oligometastatic CRPC patients. The combination of targeted therapy for macroscopic metastases with radiopharmaceuticals targeting micrometastasis is expected to be a promising new therapeutic strategy for patients with oligometastatic CRPC confined to bone.

*Trial registration* Japan Registry of Clinical Trials (jRCT) (jRCTs031200358); Registered on March 1, 2021, https://jrct.niph.go.jp/latest-detail/jRCTs031200358

**Supplementary Information:**

The online version contains supplementary material available at 10.1186/s12894-023-01202-z.

## Background

In recent years, the significance of metastasis-directed therapy for oligometastatic prostate cancer has been widely discussed [1]. The phase II STOMP trial suggests that metastasis-directed therapy of oligometastatic recurrence after radical therapy may prolong androgen deprivation therapy-free survival in metastatic hormone-sensitive prostate cancer [2]. In the ORIOLE trial, a randomized phase 2 clinical trial, men with oligometastatic prostate cancer who received stereotactic ablative radiotherapy (SABR) were significantly less likely to experience disease progression than those who opted for observation alone, and the side effects associated with SABR were mild [3]. Furthermore, recent advances in next-generation imaging, such as PET-CT/MRI with prostate-specific membrane antigen and whole-body MRI with diffusion-weighted MRI (WB-DWI) have made it possible to identify active lesions with accuracy and have led to the development of multidisciplinary treatment options for castration-resistant prostate cancer (CRPC); several studies have also reported the feasibility of targeted therapy to progressive sites, although there have been no randomized controlled trials to date [4–9]. In these reported series, the prostate specific antigen (PSA) progression-free survival with targeted therapy for oligometastatic CRPC was 8.5–17.9 months. [5–9] In some patients, however, PSA progression was reported after targeted therapy for oligometastatic CRPC, and active metastatic disease spread to multiple sites, suggesting that the limited response to targeted therapy for oligometastatic CRPC in such patients is due in part to the presence of multiple micrometastatic lesions that were not detected by imaging studies [10]. Yet in patients with oligometastatic CRPC with only bone metastases, new lesions appearing at the time of recurrence after targeted therapy tend to be limited to bone only [10]. Therefore, the combination of targeted therapy with systemic treatment for micro-bone metastases is expected to enhance the therapeutic effect.

Radium-223 dichloride (radium-223) is a radiopharmaceutical with limited tissue penetrance (∼100 µm) that selectively binds to areas of increased bone turnover and induces DNA double-strand breaks in adjacent tumor cells by emitting alpha rays, thereby inhibiting tumor growth [11–13]. Radium-223 has been shown to significantly prolong overall survival in symptomatic CRPC patients with bone metastases in the ALSYMPCA study, a phase III randomized trial. [14] In-vivo analysis has shown that radium-223 induces direct lethality in tumor subregions adjacent to bone and is expected to be effective in treating bone micrometastases, although it is relatively ineffective at controlling large tumors [15]. This therapeutic effect is consistent with the short effective radius of alpha particles, suggesting that small bone metastatic tumors may be susceptible to radium-223 [11, 15]. Therefore, it is hypothesized that the use of radium-223 in addition to targeted radiation therapy for oligometastatic CRPC confined to bone will enhance the therapeutic effect.

In this phase II, randomized trial of *Me*tastasis-*D*irected therapy with *AL*pha emitter radium-223 in men with oligometastatic CRPC (MEDAL), we will evaluate the efficacy of radium-223 in combination with metastatic site-directed radiation therapy in patients with oligometastatic CRPC whose metastases are confined to bone with progression-free survival of bone metastases on imaging as the primary efficacy endpoint.

## Methods/design

### Trial design

This study is designed as a randomized, open-label, phase II, multicenter (Tokyo Medical and Dental University, Tokyo Metropolitan Cancer and Infectious Diseases Center Komagome Hospital, Cancer Institute Hospital of JFCR, Saitama Cancer Center, Osaka International Cancer Institute, and Kanazawa University) trial in Japan. Patients will be randomized 1:1 into two groups in order to evaluate the efficacy of radium-223 in addition to targeted radiation therapy for active bone metastatic lesions in combination with best standard of care (BSoC) in patients with oligometastatic CRPC whose metastases are confined to bone, with radiological progression-free survival to exacerbation of bone metastases on WB-DWI as the primary endpoint. The study protocol follows the Standard Protocol Items: Recommendations for Interventional Trials (SPIRIT: see Additional file 1). This trial is the specified clinical trial contributed by Clinical Trials Act, thus the protocol has been reviewed and approved by Tokyo Medical and Dental University Certified Clinical Research Review Board Tokyo Medical and Dental University specially certified committee (approval No. NR2020-005). The protocol has also been reviewed and approved by the director of each participating institution and registered with the Japan Registry of Clinical Trials (jRCT) on March 1, 2021 (registry number jRCTs031200358) [16].Any important changes in the protocol, such as changes in eligibility criteria, results, or analyses, will be registered with the jRCT and communicated to the investigators, study participants, and study registries after approval by the Tokyo Medical and Dental University Certified Clinical Research Review Board Tokyo Medical and Dental University specially certified committee.

### Participants

The following inclusion criteria will be used for this trial:

1. Patients who have been diagnosed with prostate cancer either histologically or cytologically.
2. Patients who have known castration resistance, defined as follows:
  Serum testosterone ≤ 50 ng/dL (1.7 nmol/L)
  Bilateral orchiectomy or luteinizing hormone-releasing hormone (LHRH) agonist or LHRH antagonist or androgen deplevation therapy during the study period
  A new or exacerbated lesion as seen on imaging.

(3) Patients with defined active metastases localized in one to three bones as seen on WB-DWI within the last three months. Active metastatic will be assessed based on MET-RADS-P (METastasis Reporting and Data System for Prostate Cancer), which proposes to standardize data interpretation and reporting of WB-DWI performed in men with advanced prostate cancer [17].
(4) Patients aged over 20 years.
(5) Patients whose Eastern Cooperative Oncology Group Performance Status(ECOG-PS) is 0 or 1.
(6) Patients with laboratory test values consistently meeting the following cutoffs:
  Absolute neutrophils ≥ 1.5 × 10^9^/L.
  Platelets ≥ 100 × 10^9^/L.
  Hemoglobin ≥ 10.0 g/dL (6.2 nmol/L).
  Total bilirubin ≤ 1.5 times upper limit of normal (ULN).
  Aspartate aminotransferase and alanine aminotransferase ≤ 2.5 times ULN.
  Creatinine ≤ 1.5 times ULN.
  Albumin ≥ 25 g/L.
  Corrected calcium ≥ 8.4 mg/dL.
(7) Patients who are willing and able to follow the study protocol for this study, including follow-up surveys and tests.
(8) Patients who provide written informed consent for this clinical study.

The following exclusion criteria will be used for this trial:

1. Patients who have received other research drugs within the past four weeks or who are planning to receive other research drugs during the research drug administration period.
2. Patients who have received cytotoxic chemotherapy within the last three weeks or who are scheduled to receive cytotoxic chemotherapy during the study drug administration period, or patients who have not recovered from adverse events caused by cytotoxic chemotherapy given four or more weeks previously (excepting ongoing neuropathy, which is acceptable).
3. Patients who have received half-body external beam radiation.
4. Patients who have received systemic administration of radioactive isotopes of yttrium-90, lutetium-177, bismuth 213, strontium-89, samarium-153, rhenium-186, and rhenium-188 for the treatment of bone metastasis within the past 24 weeks.
5. Patients who have been treated with radium-223.
6. Patients who have received blood transfusion or erythropoietin administration within the past four weeks.
7. Patients with organ metastasis, lymph node metastasis, or active lesion in the prostate detected by WB-DWI within the past three months.
8. Patients who are in a state of urgency as indicated by clinical findings or MRI or patients who have obvious spinal cord compression.
9. Patients with other serious illnesses or medical conditions such as, but not limited to:
  Poorly controlled infectious disease.
  Heart failure of grade III or IV according to the cardiac function classification established by the New York Heart Association
  Crohn's disease or ulcerative colitis.
  Bone marrow dysplasia syndrome.

(10) Patients with fecal incontinence that is difficult to manage.
(11) Patients expected to require the use of chemotherapy or androgen receptor axis-targeted therapy (ARAT) including enzalutamide, abiraterone acetate and prednisolone during administration of the research drug.
(12) Patients with a history of irradiation to the planned irradiation site of the target therapy.

### Study population and recruitment

In accordance with the inclusion and exclusion criteria, we will enroll patients with oligometastatic CRPC whose bone metastases are localized at one to three sites as seen on WB-DWI. In addition to genuine oligometastatic CRPC patients without a history of polymetastatic disease, induced oligometastatic CRPC patients with a history of polymetastatic disease are also eligible for this trial as long as they currently have a limited number of active lesions due to previous treatments. The number of metastases and their locations in each patient are assessed through WB-DWI when disease progression is suspected before a new line of anticancer therapy is started. Systemic therapy for prostate cancer, including ARAT and chemotherapy, is prohibited during the study period except for ADT, which will be continued for the duration of the study. The physician in charge obtained informed consent and written consent forms from patients prior to study enrollment. The consent form and explanatory document included additional consent provisions for the collection and use of participant data in ancillary studies. The consent forms and explanatory documents given to the participants are shown in the Additional file 2 and Additional file 3. After inclusion, patients will be randomly assigned to radiotherapy for active metastases plus radium-223 (radium-223 group) or radiotherapy for active metastases alone (control group) in a 1:1 ratio, based on the minimization method with a treatment history of ARAT (yes vs. no) and PSA-doubling time (≥ four months vs. < four months) as stratification factors, in an open-label fashion, using a computer-generated minimized randomization allocation sequence as part of the electronic data capture (EDC) system.

### Interventions

**Fig. 1:**
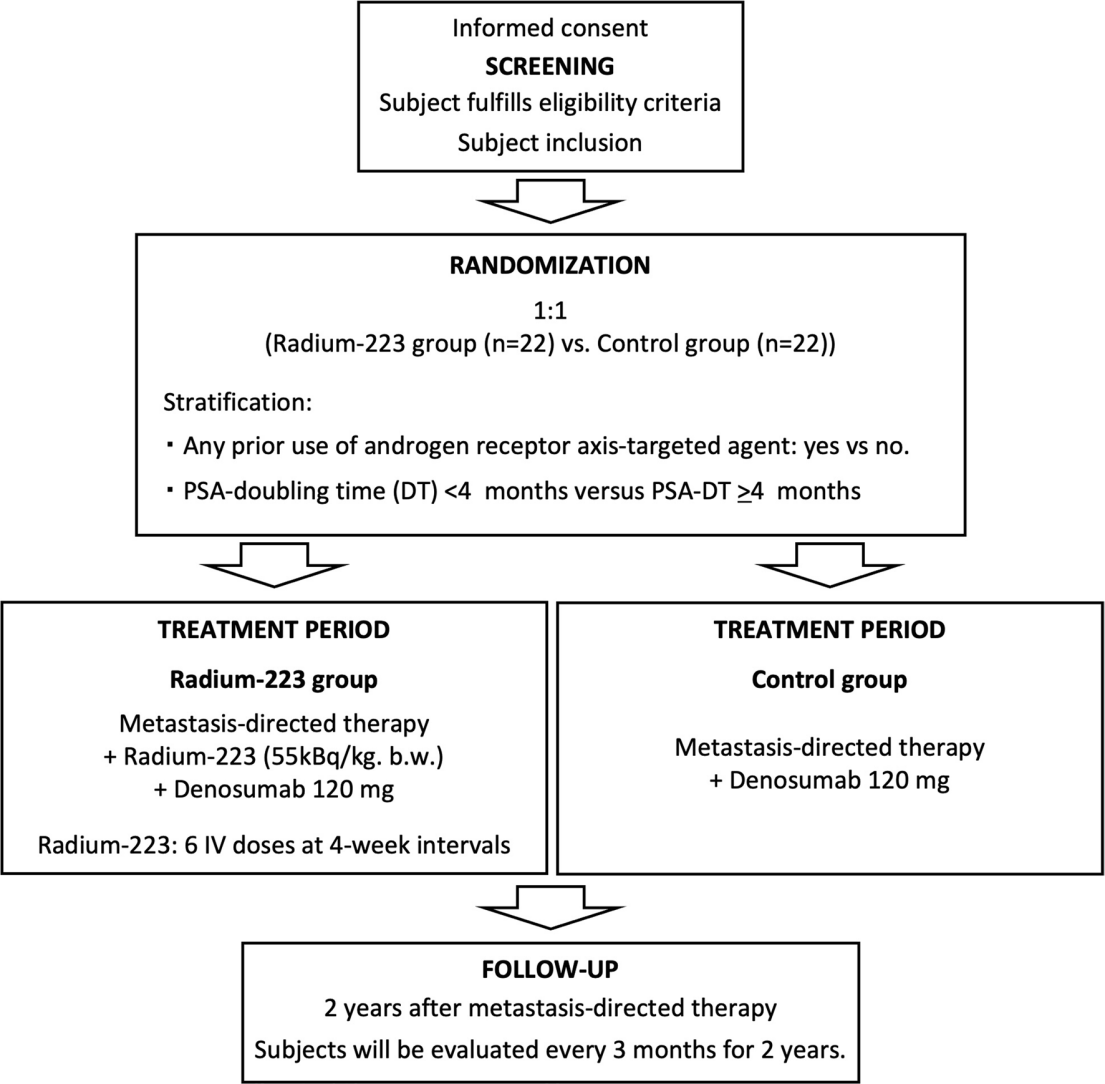
Design of the MEDAL trial

Figure 1 gives an overview of the procedures that patients will undergo during the study. In both study groups, all active metastases will be treated with stereotactic body radiation therapy or intensity-modulated radiation therapy for local control. All patients will then receive six 120-mg doses of subcutaneously injected denosumab every four weeks as the best standard of care (BSoC) for CRPC patients with bone metastasis. The control group will receive BSoC only, while the radium-223 group will also receive a maximum of six doses of the study's research drug, radium-223; Xofigo® (Bayer Inc.), intravenously at 55 kBa/kg every four weeks, starting within two weeks after the completion of radiotherapy for active metastases. No placebo will be used in this study.

If an adverse reaction occurs due to radium-223 administration, the degree of the adverse reaction should be evaluated according to Common Terminology Criteria for Adverse Events (CTCAE) version 5.0, and the dose should be withdrawn or reduced. In case of grade 3 or higher neutropenia, anemia, or thrombocytopenia, administration should be postponed until recovery to grade 2 or lower, and then resumed after confirmation of recovery. If the grade of neutropenia or thrombocytopenia does not recover to grade 2 or below within 6 weeks of the last dose, administration should be discontinued. For grade 3 or higher diarrhea, nausea, vomiting, or constipation, postpone administration until recovery to grade 2 or lower, and resume administration after confirming recovery. If grade 4 events persist for more than 7 days, administration should be discontinued.

### Follow-up, data collection, and data protection

**Fig. 2:**
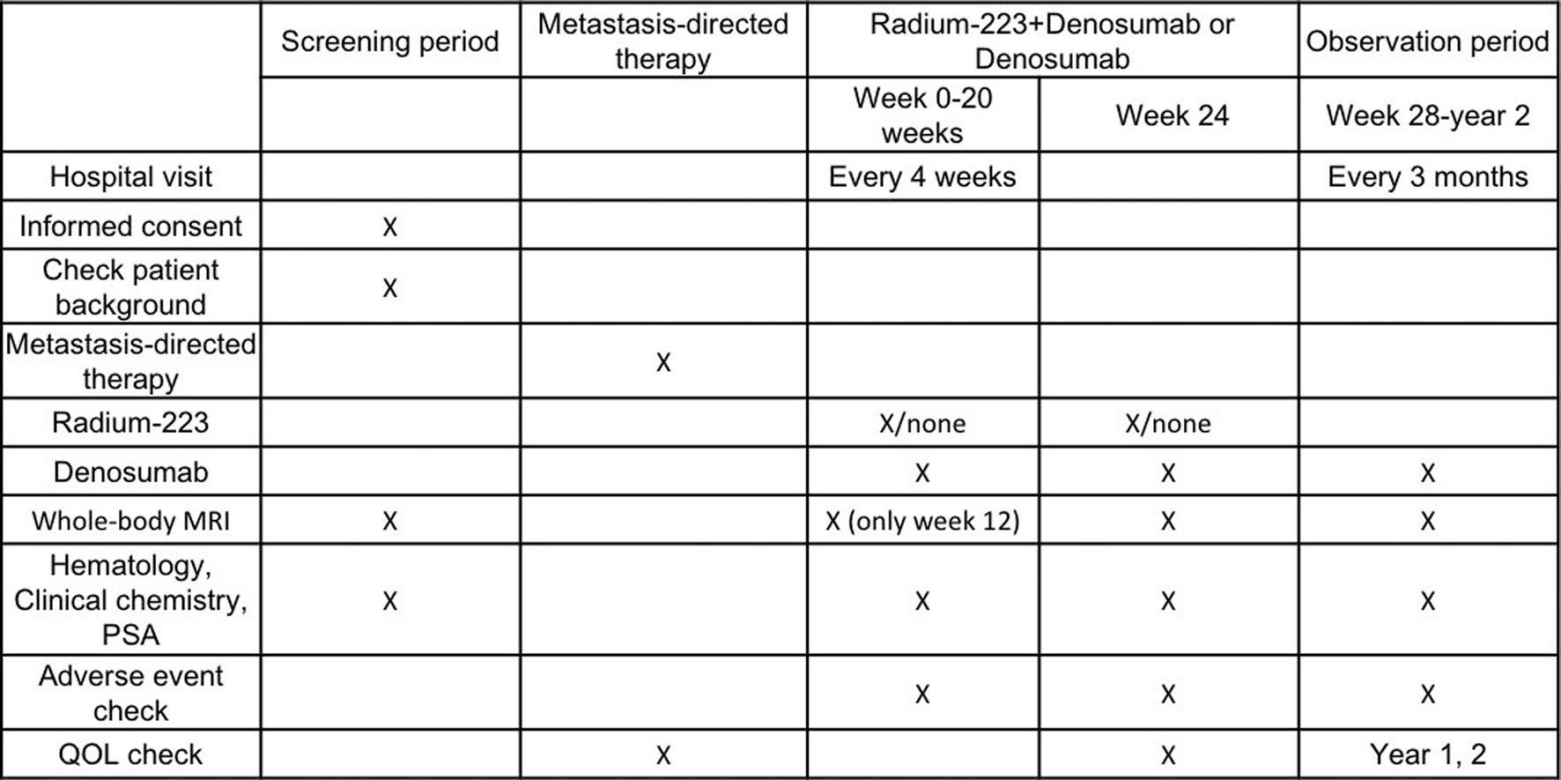
Intervention and assessment schedule for the MEDAL trial according to the Recommendations for Interventional Trials (SPIRIT). Patients will visit the hospital every 4 weeks until about 24 weeks after radiotherapy, and then every 3 months for the next 2 years to collect data. *according to the Common Toxicity Criteria for Adverse Events (CTCAE v 5.0), **questionnaires: FACT-P, EQ-5D. *PSA* Prostate-specific antigen, *QOL* Quality of life

Patients will be seen every four weeks for the six months following the end of targeted radiation therapy and every three months thereafter for the next two years, and ECOG-PS, PSA, blood counts, and biochemical tests will be evaluated at each visit. (Fig. 2) In addition, the occurrence of adverse events related to targeted therapy and radium-223 administration will be evaluated according to the CTCAE version 5.0. WB-DWI will be performed every three months from the end of targeted therapy to the study end date. Functional assessment of cancer therapy-prostate (FACT-P) and EuroQual 5 dimension (EQ-5D) will be assessed at the time of randomization, at the end of the study treatment period, and one year after the last dose of the study drug. All patients will be monitored for survival status and subsequent therapies until the two-year follow-up period has ended for the last subject enrolled in the study. To improve monitoring adherence, the physician in charge will explain the need for monitoring tests at the time of the patient's visit and encourage the patient to undergo the follow-up for study. To improve data quality, a clinical research coordinator will perform data entry from the medical record, and the data will be centrally monitored. To protect patients' personal information, a unique identification code will be assigned to every patient. All data will be protected on the EDC system as password-accessible electronic data files before, during, and after the study, and only the investigators will have access to the files.

### Outcomes

#### Primary outcome measure

Progression-free survival from randomization to progression of bone metastases on WB-DWI (Blinded independent central review (BICR)) or to death from any cause is the primary endpoint. The efficacy of treatment will be assessed based on WB-DWI results as interpreted by central judgement. Progression of bone metastases will be assessed by evaluators both inside and outside the field of radiotherapy. Evaluation will be based on the MET-RADS-P [17]. The central evaluator will be blinded to the allocation information of the study and control groups and will evaluate the results with the consent of both evaluators.

#### Secondary outcome measures

Secondary outcomes include the following:

- (1) Progression-free survival from randomization to appearance of new bone metastases outside the field of targeted therapy on WB-DWI (according to BICR).
- (2) Progression-free survival from randomization to exacerbation of bone metastases on WB-DWI (according to each investigator’s judgment).
- (3) Progression-free survival from randomization to the appearance of new bone metastases outside the field of targeted therapy on WB-DWI (according to each investigator’s judgment).
- (4) Overall survival.
- (5) Time to PSA progression.
- (6) Changes in PSA.
- (7) Time to exacerbation of total alkaline phosphatase (ALP).
- (8) Total ALP response defined as a reduction of ≥ 30% from the baseline value, confirmed ≥ 4 weeks later.
- (9) Normalization of total ALP.
- (10) Changes in total ALP.
- (11) Interval between completion of targeted therapy and start of treatment with other hormone therapy, anticancer drugs or radiation therapy.
- (12) Therapeutic effect on bone metastatic lesions as assessed by MET-RADS-P at six months after completion of targeted therapy.
- (13) Therapeutic effect on whole metastatic lesions as assessed by MET-RADS-P at six months after completion of targeted therapy.
- (14) Quality of life (QOL): Changes in FACT-P at six months and one year after completion of targeted therapy.
- (15) QOL: Changes in EQ-5D at six months and one year after the completion of targeted therapy.

### Safety endpoints

Safety endpoints include the following:

- Incidence and severity of adverse events and abnormal laboratory test values; adverse events will be classified according to the ICH International Pharmaceutical Glossary (MedDRA) version 11.0, and their severity will be classified according to the Common Terminology CTCAE version 5.0
- Incidence of serious adverse events.
- Percentage of patients who discontinue treatment due to adverse events.

### Research drug

The investigational drug is Xofigo®, its identification code is D10398, and its chemical name is radium-223 dichloride. This study is covered by clinical research insurance, and compensation may be provided in the event of side effects not included in the list of possible side effects caused by radium-223 administration.

### Determination of sample size

Because this study is a phase II exploratory clinical trial to evaluate endpoints, treatment methods, and target patient groups to be tested in a subsequent phase III trial, the target number of patients is 44 (including an anticipated 40 evaluable cases and four dropouts (10%)) based on the number of cases that can be accumulated during the study period (enrollment period: two years, observation period: two years). Assuming a median radiologic bone progression-free survival in the control and study treatment groups of six and 12 months, respectively (hazard ratio: 0.50), the power will be 54.7% with an allocation ratio of 1:1 and a two-sided significance level of 0.05. The target sample size would be achieved according to the number of cases we have treated to date.

### Statistical analysis

The analyses are based on intention-to-treat principle. The primary analysis of progression free survival is planned when approximately 90% of patients had the progression. The progression-free survivals between the two group will be compared using a stratified log-rank test at the two-sided significant level of 0.05. The hazard ratio of progression free survival is estimated by stratified Cox regression analysis. The stratification factors are the same used for randomization. The Kaplan–Meier method is used to estimate the median progression free survival. Patients with no documentation of disease progression or death during the trials are censored at date of last contact alive. The other time-to-event endpoints will be analyzed with the same methods as the progression free survival. The proportion of patients with binary endpoint and the corresponding 95% confidence intervals (CIs) for each group will be estimated by using the Clopper-Pearson method and compared between the two groups with the Cochran–Mantel–Haenszel test. In the continuous endpoints, the mean change and the corresponding 95% CI from baseline at each visit will be estimated for each group and compared between the two group with analysis of covariance (or mixed-effects model for repeated measures). No interim analysis will be conducted.

### Dissemination

The results will be submitted to a peer-reviewed journal for publication and will be presented at national and international scientific meetings.

## Discussion

We have reported the protocol for a open-label randomized study comparing metastasis-directed therapy ± radium-223 in CRPC patients with metastatic lesions in one to three bones. To the best of our knowledge, this will be the first randomized trial to evaluate the effect of radium-223 in combination with targeted therapy in oligometastatic CRPC patients. Metastatic cancers with three or fewer metastatic sites comprise 30% of CRPC cases; in 35% of these cases, the metastases were confined to bone as assessed by WB-DWI [8]. It is expected that treatment strategies integrating the systemic treatment of micrometastatic disease using bone-targeting radiopharmaceuticals with metastasis-directed therapy may emerge as a novel therapeutic option for patients with oligometastatic CRPC confined to bone.

To date, there is no evidence regarding the efficacy of radiopharmaceutical therapy against oligometastatic CRPC, but the ongoing RAVENS and RROPE trials are evaluating similar treatment strategies in patients with castration-sensitive, hormone-sensitive prostate cancer. The RAVENS trial is a Phase II study evaluating the efficacy of the combination of radium-223 and radiotherapy against active metastases in hormone-sensitive prostate cancer with one to three asymptomatic metastatic tumors of the bone or soft tissue (with at least one bone metastasis), and is scheduled to be completed in August 2024 (NCT04037358) [18]. The RROPE trial, a phase IIa, open-label, single-arm, prospective study, is evaluating the effectiveness of SABR plus radium-223 for bone metastases with attention to time to initiation of ADT in oligo-metastatic recurrent prostate cancer with fewer than six bone metastases, and is scheduled for completion in November 2022 (NCT03304418).

In CRPC, disease status may not be consistent with PSA changes: the ALSYMPCA study, which showed that radium-223 prolonged overall survival, did not confirm an association between treatment response and PSA changes [19]. In this study, therefore, we plan to evaluate the efficacy of radium-223 in combination with targeted therapy using WB-DWI, which can monitor the therapeutic efficacy and activity of bone metastases, with radiological bone PFS as the primary endpoint [20]

Because this is an open-label RCT with unblinded participants and therapists, there is potential for selective bias. Because the primary outcome, radiographic PFS of bone, will be assessed by blinded central radiologists, however, any findings related to the primary outcome will be less prone to assessment bias.

## Conclusion

This protocol describes the design of a randomized, open-label, phase II, multicenter trial that will evaluate the efficacy of radium-223 in combination with metastatic site-directed radiation therapy in patients with oligometastatic CRPC whose metastases are confined to bone. It is hoped that the results of this study will provide a sound basis for a prospective, randomized phase III trial.

## Supplementary Information

**Additional file 1: **SPIRIT 2013 Checklist for the MEDAL trial.

**Additional file 2: **The explanatory document translated to English (the original document is written in Japanese).

**Additional file 3: **The consent form given to participants.

## Data Availability

Data sharing is not applicable to this article because the current study is still open for inclusion of patients.

## Abbreviations

ALP: Alkaline phosphatase
ARAT: Androgen receptor axis-targeted therapy
BICR: Blinded independent central review
BSoC: Best standard of care
CRPC: Castration-resistant prostate cancer
CTCAE: Common terminology criteria for adverse events
DWI: Diffusion-weighted MRI
ECOG-PS: Eastern cooperative oncology group performance status
EDC: Electronic data capture
EQ-5D: EuroQual 5 dimension
FACT-P: Functional assessment of cancer therapy-prostate
jRCT: Japan registry of clinical trials
LHRH: Luteinizing hormone-releasing hormone
MET-RADS-P: METastasis reporting and data system for prostate cancer
PSA: Prostate specific antigen
QOL: Quality of life
SABR: Stereotactic ablative radiotherapy
Radium-223: Radium-223 dichloride
SPIRIT: Standard protocol items: recommendations for interventional trials
ULN: Upper limit of normal
WB-DWI: Whole-body MRI with diffusion-weighted MRI
