# Supplementary material for: A phase II randomized trial of metastasis-directed therapy with alpha emitter radium-223 in men with oligometastatic castration-resistant prostate cancer (MEDAL)
Source: BMC Urol. 2023 Mar 6;23:33. doi: 10.1186/s12894-023-01202-z (PMC9987040; doi:10.1186/s12894-023-01202-z)
Supplement: Supplementary file 2 — Additional file 2: The explanatory document translated to English (the original document is written in Japanese). [file 12894_2023_1202_MOESM2_ESM.docx]

**Explanatory document**

**“A phase II randomized trial of metastasis-directed therapy with alpha emitter radium-223 in men with oligometastatic castration-resistant prostate cancer”**

**1. Introduction**

It is the mission of a university hospital to establish new treatment methods through clinical research, which can be accomplished with the cooperation of patients. The clinical study in which you are requested to participate this time is a “A phase II randomized trial of metastasis-directed therapy with alpha emitter radium-223 in men with oligometastatic castration-resistant prostate cancer.” The study will be conducted by physicians involved in actual medical treatment in light of medical necessity and importance. This is not a clinical trial conducted by a pharmaceutical company to investigate the safety and usefulness of a new drug and obtain approval from the Ministry of Health, Labour and Welfare. However, this research has been approved by the hospital director as the administrator of the medical institution based on the opinion of the Clinical Research Review Committee approved by the Ministry of Health, Labour and Welfare, and the implementation plan has been submitted to the Minister of Health, Labour and Welfare. It is up to you to decide whether you wish to participate in the research. If you do not participate in the research, you will not be disadvantaged in any way, including medical care. You may also withdraw at any time during your participation.

Your disease is castration-resistant prostate cancer confined to one to three bone sites, and in recent years, radiation therapy for bone metastases has been effective in inhibiting disease progression for about 7 months. However, when the disease progresses, progression often appears at multiple sites, and it is assumed that microlesions undetected by imaging studies are already present. Intravenous radium-223, a drug approved in Japan for the treatment of castration-resistant prostate cancer metastasized to the bone, contains a radioactive substance called Xofigo®, which emits radiation called alpha rays. Radium-223 has the same tendency to accumulate in bones as calcium, a component of bones. When administered as an injectable drug, it is transported to bone metastatic sites where metabolism is active, and the alpha rays emitted from these sites are expected to suppress the growth of prostate cancer with bone involvement. Therefore, in addition to radiation therapy for bone metastases identified by imaging tests, radium-223 therapy for microlesions not detected by imaging tests is expected to have a better effect of inhibiting disease progression.

Therefore, this study is designed to investigate the effectiveness of combining radiation therapy with radium-223 treatment for bone metastases. If your symptoms and disease background are consistent with this study designed to investigate the use of radium-223 in combination with radiotherapy, we would like to confirm your willingness to participate in the study. Please note that even if you agree, you may not be able to participate in the study depending on your examination findings.

Radium-223 has already been used as a drug for castration-resistant prostate cancer metastasized to the bones and has resulted in prolonged survival of approximately 3 months. Conversely, its side effects included anemia (19%), thrombocytopenia (7%), and leukopenia (3%). The combination of radiotherapy and radium-223 may be effective for your disease, and if it is shown to be effective and safe in this study, it may become widely used in patients with the same medical condition as yours.

In this study, subcutaneous injection of denosumab, called RANMARK^®^ will be used as a prophylactic agent against bone metastases. Subcutaneous injection of denosumab has been used for bone metastasis of prostate cancer as a preventive agent against the appearance of symptoms and surgical treatment of bone metastasis. However, hypocalcemia and osteonecrosis of the jaw occur in approximately 6% and 2% patients, respectively.

This study is funded by a research grant from Bayer Corporation, the manufacturer and distributor of Xofigo®. Before accepting the research grant for this study, a declaration was made to the Conflict of Interest Committee for Clinical Research at the School of Medicine, and it was judged that the acceptance of the research grant is unlikely to bias the results of this study, and the implementation of the study has been approved by the hospital director.

**2. Purpose of this study**

Currently, the efficacy of radiation therapy for bone metastases in castration-resistant prostate cancer confined to one to three bones is approximately 7 months, and a treatment strategy to improve the efficacy is needed. Intravenous radium-223 is expected to be effective in treating bone metastases that are not detected by imaging. In addition to radiotherapy for bone metastases identified on imaging, this study will confirm the efficacy and safety of radium-223 treatment for micrometastases that are not detected by imaging, which is very significant for the treatment of castration-resistant prostate cancer confined to one to three bones.

**3. Methods of this study**

To correctly judge the efficacy of the combination of radiotherapy and radium-223 for bone metastases, the patients participating in the study will be divided into two groups—one group will receive radiotherapy only for bone metastases and the other group will receive radiotherapy plus radium-223 for bone metastases. The group assignment will not be based on your physician’s judgment or your wishes, but it will be done mechanically for objective evaluation. The probability of being in each group is 50%.

The effect of treatment will be evaluated every 3 months based on findings of whole-body magnetic resonance imaging (MRI) and blood tests, including those of measuring prostate-specific antigen (PSA) levels. Your test results obtained in this study will be used to evaluate your condition. We will also use the test information obtained prior to your consent to participate in this study. Your name, test information, and other personal information will be kept strictly confidential and anonymized so that your information will not be identifiable throughout the study. The information will be stored in a password-protected electronic file for 10 years after the completion of the study (university regulations) by Soichiro Yoshida, who will be responsible for storing the information; at the time of disposal, the information will be disposed of in such a way that your personal information cannot be identified.

If you are currently undergoing treatment another hospital, please let us know the name of the hospital, the name of the disease, and the medication you are taking. This is important for ensuring the safety of the study. If you are a patient at another hospital, please understand that we may inform the hospital of your participation in this study.

**Flowchart of the tests**

**
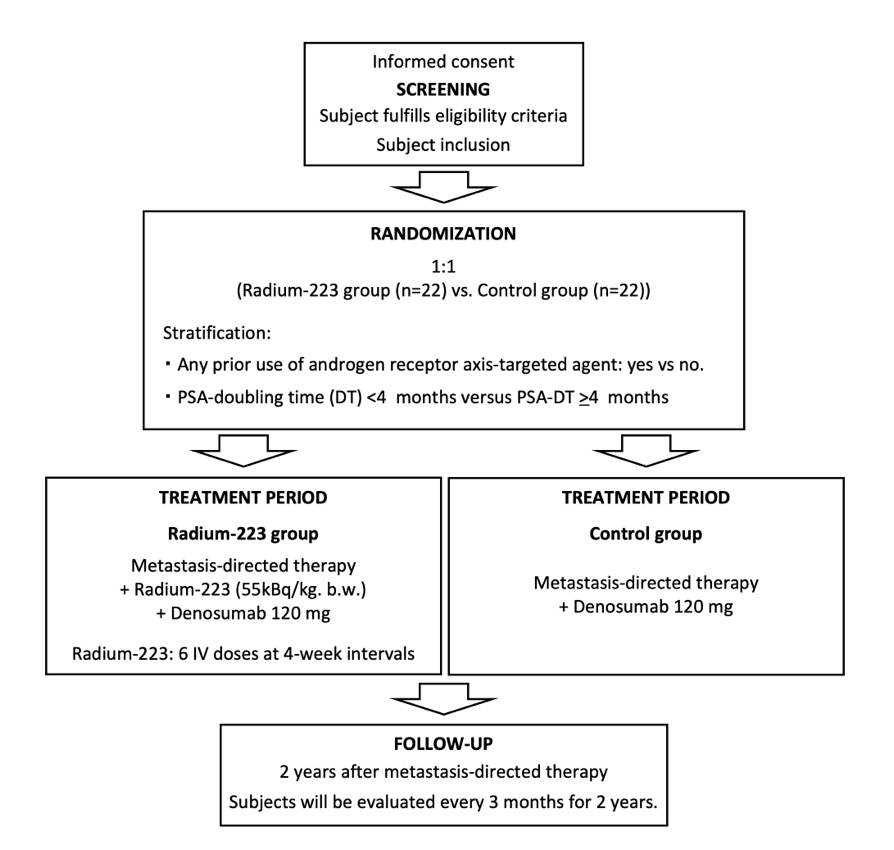
**

The flow chart of this study is shown above.

**Schedule**


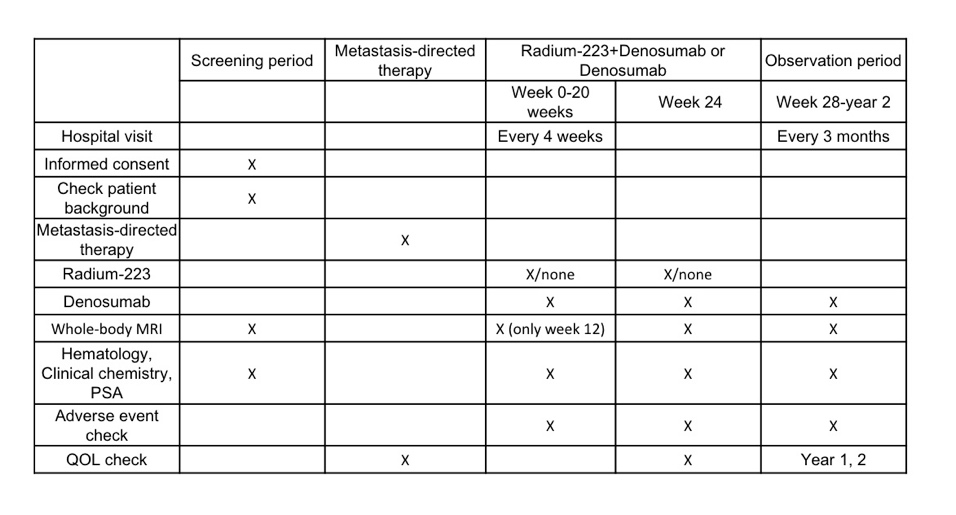


- Hematological tests and blood chemistry tests are performed to confirm safety. Blood chemistry tests include the PSA test. This is to confirm the effectiveness of the treatment.
- Adverse events are all events that are undesirable to health, such as side effects, regardless of whether they are causally by the medication or radiation therapy.

**4. Eligibility for this study**

Patients are eligible for this study if they meet all the following criteria:

(1) Histologically or cytologically confirmed prostate cancer

(2) Known castration resistance defined as follows:

- - - Serum testosterone level ≤50 ng/dL (1.7 nmol/L)
    - Bilateral vasectomy or maintenance of luteinizing hormone-releasing hormone (LHRH) agonist, LHRH antagonist therapy, or androgen deprivation therapy during the study period
    - Exacerbation of serum PSA level (two consecutive increases in PSA level from the historical reference value at ≥1-week intervals or an increase of ≥1 ng/dL from the lowest PSA value)
    - Patients with new or worsening lesions on imaging tests

(3) Patients with one to three active metastases confined to the bone on whole-body MRI within the past 3 months

(4) Patients aged >20 years

(5) Patients with Eastern Cooperative Oncology Group Performance Status score of 0–1

(6) Patients meeting the following clinical criteria:

- - - Absolute neutrophil count ≥1.5 × 10^9^/L
    - Platelet count ≥100 × 10^9^/L
    - Hemoglobin level ≥10.0 g/dL (6.2 nmol/L)
    - Total bilirubin level ≤1.5 times the upper limit of institutional standard (ULN)
    - Aspartate aminotransferase and alanine aminotransferase levels ≤2.5 times the ULN
    - Creatinine level ≤1.5 times of ULN
    - Albumin level ≥25 g/L
    - Corrected calcium level ≥8.4 mg/dL

(7) Patients who are willing and able to comply with the study protocol, including follow-up visits and examinations

(8) Patients who have received sufficient explanation of the information about this study and signed the consent form

Patients are not eligible for this study if they meet all the following criteria:

(1) Patients who have received other research drugs within the past 4 weeks or are scheduled to receive other research drugs during the period of drug administration

(2) Patients who have received cytotoxic chemotherapy within the past 3 weeks, those are scheduled to receive cytotoxic chemotherapy during the study drug administration period, or those who have not recovered from an adverse event due to cytotoxic chemotherapy administered prior to 4 weeks (ongoing peripheral neuropathy is acceptable)

(3) Patients who have received external hemisphere irradiation

(4) Patients who have received systemic administration of radioactive isotopes such as yttrium-90, lutetium-177, bismuth-213, strontium-89, samarium-153, rhenium-186, or rhenium-188 for the treatment of bone metastases within the past 24 weeks

(5) Patients treated with radium-223

(6) Patients who have received blood transfusion or erythropoietin within the past 4 weeks

(7) Patients with organ or lymph node metastasis or active disease in the prostate on whole-body MRI within the past 3 months

(8) Patients with clinical or MRI findings of urgency or obvious spinal cord compression

(9) Patients with other serious diseases or medical conditions listed below (but not limited to):

- - - Uncontrolled infections
    - Patients with other serious diseases or medical conditions (but not limited to): uncontrolled infections, New York Heart Association heart failure (III or IV)
    - Crohn’s disease or ulcerative colitis
    - Myelodysplastic syndromes

(10) Patients with fecal incontinence that is difficult to manage

(11) Patients who are judged to require f new hormonal agents including olaparib, enzalutamide, abiraterone acetate, and prednisolone or chemotherapy while receiving the study drug

(12) Patients with a history of prior irradiation at the planned target therapy site

(13) Patients diagnosed with organ metastasis in the past

**5. Expected duration of participation in this study**

If you participate in this study, your expected participation period will be until March 31, 2025. The expected duration of participation in this study is 14 weeks for the pre-observation period, 24 weeks for the study drug administration period, and 72 weeks for the post-observation period, accounting for a total of 110 weeks.

**6. Number of patients expected to participate in this study**

This study will be conducted at four hospitals in Japan, and 44 patients are expected to participate.

**7. Anticipated benefits and possible side effects of this study drug**

A total of 614 patients with castration-resistant prostate cancer with bone metastases have been treated with radium-223; they had increased survival by approximately 3 months compared to patients who did not receive the drug. In addition, 600 patients with castration-resistant prostate cancer with bone metastases were treated with radium-223, and the most common side effects reported were nausea (21%), anemia (18%), diarrhea (17%), bone pain (16%), and fatigue (12%). The other reported incidences include myelosuppression: neutropenia (3.9%), thrombocytopenia (7.4%), anemia (19.3%), leukopenia (3.2%), lymphopenia (2.0%), and pancytopenia (1.7%).

If you have any of the following symptoms, please consult your doctor:

*Symptoms of anemia*

Feeling of fatigue, headache, dizziness, ringing in the ears, shortness of breath, palpitations, or shortness of breath when climbing stairs or hills

*Symptoms of low platelet count*

Nose bleeding, bleeding of gums, bruising, subcutaneous bleeding, or difficulty in stopping bleeding

*Symptoms of decreased neutrophil, white blood cell, and lymphocyte counts*

Fever or sore throat

**8. Other treatment options if you do not participate in this study**

Even if you do not participate in this study, there are conventional ways to treat your disease, and your doctor will choose the appropriate treatment for you, taking your wishes into consideration. Specifically, systemic treatments are available, such as olaparib, chemotherapy with docetaxel and cabazitaxel, hormone therapy with enzalutamide and abiraterone, and radium-223 therapy without concomitant radiation therapy.

Patients who have not previously received systemic treatments such as olaparib, chemotherapy with docetaxel or cabazitaxel and hormonal therapy with enzalutamide and abiraterone may be able to receive these treatments after the completion of this study.

**9. In case of any damage to your health during this study**

This study has been scientifically planned and will be carefully conducted based on previous reports. If you experience any side effects or other health problems during or after the clinical study, your doctor will provide you with appropriate medical attention and treatment. Since this study will be conducted using medications that are already available in the market within their indications, any health problems caused by these medications will be treated in the same way as normal medical treatment. In addition, if a certain amount of health damage occurs due to side effects, the patient may be eligible for benefits under the Adverse Drug Reaction Relief System.

This study is covered by clinical research insurance, and compensation may be provided in the event of side effects not included in the list of possible side effects caused by radium-223 administration.

**10. Participation in this study is voluntary**

Participation in this study is voluntary. You may withdraw your consent at any time, even after you have participated. Even if you do not participate or revoke your consent to participate, we will provide the most appropriate treatment for you, and you will not be treated adversely or suffer any disadvantage in treatment.

**11. Information regarding the use of radium-223 in combination with radiation therapy will be communicated to you as needed**

If we obtain any information that may affect your family’s opinion, we will inform you as soon as possible and ask for your consent to continue the study. If you do not give your consent, you will still be able to receive other treatments. If you want to know more about the study and its methods, please let the principal investigator know. To the extent that it does not interfere with our research, you will be given access to materials related to the protocol and methods.

**12. We may discontinue the use of radium-223 in some cases**

In case of side effects caused by radium-223 administration, the dose will be reduced or withdrawn according to the following criteria. The degree of side effects will be evaluated in accordance with the Common Terminology Criteria for Adverse Events, version 3.0.

| Side effects | Treatment |
| --- | --- |
| Grade ≥3 neutropenia, anemia, or thrombocytopenia | Administration should be postponed until recovery to Grade ≤2 and then resumed after confirmation of recovery. If the grade does not recover to ≤2 within 6 weeks from the last dose, administration should be discontinued. |
| Grade ≥3 diarrhea, nausea, vomiting, or constipation | Dosing should be postponed until recovery to Grade ≤2 and then resumed after confirmation of recovery. |
| Grade 4 or other events | If any of these events persists for >7 days, discontinue administration. |

**13. If you participate in this study, your medical records and other information may be examined during or after the study**

To confirm that this study is being conducted properly while protecting the human rights of patients, your medical records may be reviewed by persons involved in this research (such as hospital staff; review committees; Ministry of Health, Labour and Welfare personnel; monitoring personnel; and auditors). In such cases, personal information will be handled appropriately. By signing the consent form, you are giving permission for access.

**14. Even if the results of this study are made public, your identity will not be revealed**

The results obtained in this study will be registered in the Japan Registry of Clinical Trials (jRCT: https://jrct.niph.go.jp/) of the Ministry of Health, Labour and Welfare and may also be published in medical journals, websites of universities, academic societies, or other platforms. However, your name and other personal information will not be revealed. For example, we will use a different code number that cannot be inferred from your name or initials; therefore, your privacy will be protected. The samples and information obtained in this study will be strictly controlled and stored, but they may be used in the future or provided to other research institutions. For example, we may want to reanalyze your samples due to new discoveries or we may want to aggregate the information with data from a larger number of patients. In such cases, when a new research plan is formulated, the Tokyo Medical and Dental University Clinical Research Review Committee will be consulted for approval, and you will be notified of the details of the research.

**15. If you agree to participate in this research, please observe the following points**

When visiting other departments or hospitals or when purchasing medicines at pharmacies, please be sure to inform the doctor or pharmacist concerned that you are participating in the research and consult with the doctor in charge of the research in advance whenever possible. When this study is completed, a normal insurance-covered treatment will be provided if necessary. If you wish to continue to receive outpatient consultations after the completion of this study, please inform the principal investigator.

In addition, because of the nature of the drug, the injection date cannot be changed. Please consult your doctor carefully before deciding on your schedule.

The alpha rays emitted from radium-223 only affect <0.1 mm of the patient’s body; hence, there is almost no radiation effect on the patient’s family or other people who contact with the patient. However, for about 1 week after radium-223 injection, a small amount of radium-223 may remain in the blood or stool. Therefore, patients and their family members (caregivers) should observe the following precautions:

*Daily life precautions*

1. If the patient experiences bleeding, wipe up the blood with toilet paper and flush it down the toilet.

2. Wear disposable rubber gloves before handling the patient’s urine or stool or touching clothing soiled with these substances.

3. If you contact with the patient’s blood or other bodily fluids on your hands or skin, immediately wash the affected area thoroughly with soap and water.

4. Try to refrain from sexual activity. In addition, use proper birth control during treatment with this medicine and 6 months after treatment discontinuation.

5. Minimize contact between the patient and children or pregnant women for 2–3 days after of Radium-223 injection. (Avoid carrying children for a long time).

6. When patients take a bath, they should do so at the end of the day. After bathing, brush the bathtub with detergent and wash thoroughly.

*Cautions for handling laundry*

1. Wash clothes worn by patients separately from other people’s clothes.

2. Prewash sheets and underwear that have blood or urine on them before washing them.

*When defecating, urinating, or vomiting*

1. Sit on the toilet seat to urinate.

2. After using the toilet, flush the toilet about two times.

3. If urine or stool is spilled on the toilet bowl or floor, wipe up with toilet paper and flush the toilet.

4. Wash your hands well with soap and water after urinating or defecating.

5. If the patient’s feces or vomit contact with your hands or skin, wash them immediately with soap and water thoroughly.

*Precautions for patients using diapers and urinary catheters*

1. Wear disposable gloves when handling a patient’s diaper, urinary catheter, or urine storage pack. (Diapers should be sealed in a plastic bag to prevent leakage of the contents and disposed of as general waste.)

2. Patients with urinary incontinence and those using diapers should use plastic sheets.

3. Urinary drainage or urine in urine packs used with catheters should be disposed of in the toilet and flushed with water twice, and hands should be washed thoroughly after disposal.

**16. About your cost burden**

Radiotherapy and drug administration in this study will be performed within the scope of normal insurance coverage as the use of over-the-counter drugs is within the scope of this study.

Whole-body MRI examinations to evaluate the effects of treatment will be performed every 3 months in this study. Whole-body MRI examinations performed in addition to the semiannual imaging examinations performed in regular medical care will be covered by the research expenses of this study. This is equivalent to half of the total cost of whole-body MRI. This study is funded by Bayer Corporation, which manufactures and sells radium-223, but there is no conflict of interest.

**17. These doctors will take care of you**

- Tokyo Medical and Dental University

(Principal investigator) Soichiro Yoshida, Department of Urology (ext. 5295).

(Subinvestigator) Yasuhisa Fujii, Department of Urology (ext. 5295)

(Subinvestigator) Ryoichi Yoshimura, Department of Radiation Therapeutics and Oncology (ext. 5311)

(Subinvestigator) Yo Matsuoka, Department of Urology (ext. 5295)

(Subinvestigator) Minato Yokoyama, Department of Urology (ext. 5295)

(Subinvestigator) Hajime Tanaka, Department of Urology (ext. 5295)

(Subinvestigator) Sho Uehara, Department of Urology (ext. 5295)

At night (5:00–9:00 p.m.) and on holidays, please call the emergency room (03-3813-6111) and ask for the urologist on duty.

- Tokyo Metropolitan Cancer and Infectious Diseases Center Komagome Hospital

(Principal investigator) Fumitaka Koga, Department of Urology (ext. 4021)

(Subinvestigator) Masaya Ito, Department of Urology (ext. 4021)

(Subinvestigator) Sara Hayakawa, Department of Radiation Oncology (ext. 4323)

At night (5:00–9:00 p.m.) and on holidays, please call the emergency room (03-3823-2101) and ask for the doctor on duty in the Department of Nephrology and Urology.

- Cancer Institute Hospital, Japanese Foundation for Cancer Research

(Principal investigator) Junji Yonese, Department of Genitourinary Oncology (ext. 7094)

(Subinvestigator) Yasuo Yoshioka, Department of Radiation Oncology (ext. 8033)

(Subinvestigator) Takeshi Yuasa, Department of Genitourinary Oncology (ext. 7449)

(Subinvestigator) Noboru Numao, Department of Genitourinary Oncology (ext. 7619)

(Subinvestigator) Yoshinobu Komai, Department of Genitourinary Oncology (ext. 7095)

(Subinvestigator) Tomohiko Oguchi, Department of Genitourinary Oncology (ext. 7611)

At night (5:00–9:00 p.m.) and on holidays, please call the emergency room (03-3520-0111) and ask for the urologist on duty.

- Saitama Cancer Center

(Principal investigator) Yukio Kageyama, Department of Urology (ext. 5003)

(Subinvestigator) Masaharu Inoue, Department of Urology (ext. 5163).

(Subinvestigator) Yasumasa Shimano, Department of Radiation Oncology (ext. 5240)

(Subinvestigator) Shigehiro Kudo, Department of Radiation Oncology (ext. 5198)

(Subinvestigator) Hiroki Ushijima, Department of Radiation Oncology (ext. 5194)

At night (5:00–9:00 p.m.) and on holidays, please call the emergency room (048-722-1111) and ask for the urologist on duty.

- Osaka International Cancer Institute

(Principal investigator) Akira Nagahara, Department of Urology (ext. 5094)

(Subinvestigator) Kazuo Nishimura, Department of Urology (ext. 5094)

(Subinvestigator) Katsuyuki Nakanishi, Department of Diagnostic and Interventional Radiology (ext. 5094)

(Subinvestigator) Koji Konishi, Department of Radiation Oncology (ext. 5094)

At night (5:30–9:00 p.m.) and on holidays, please call the emergency room (06-6945-1181) and ask for the urologist on duty.

- Kanazawa University Hospital

(Principal investigator) Atsushi Mizokami, Department of Urology (ext. 2393)

(Subinvestigator) Yoshihumi Kadono, Department of Urology (ext. 2393)

(Subinvestigator) Kouji Izumi, Department of Urology (ext. 2393)

(Subinvestigator) Kazuyoshi Shigehara, Department of Urology (ext. 2393)

(Subinvestigator) Takahiro Nohara, Department of Urology (ext. 2393)

(Subinvestigator) Shohei Kawaguchi, Department of Urology (ext. 2393)

(Subinvestigator) Masashi Ijima, Department of Urology (ext. 2393)

(Subinvestigator) Hiroshi Yaegashi, Department of Urology (ext. 2393)

(Subinvestigator) Hiroaki Iwamoto, Department of Urology (ext. 2393)

(Subinvestigator) Suguru Kadomoto, Department of Urology (ext. 2393)

(Subinvestigator) Yuki Kato, Department of Urology (ext. 2393)

(Subinvestigator) Taito Nakano, Department of Urology (ext. 2393)

(Subinvestigator) Tomomi Nakagawa, Department of Urology (ext. 2393)

(Subinvestigator) Hiroshi Kano, Department of Urology (ext. 2393)

(Subinvestigator) Takafumi Shimada, Department of Urology (ext. 2393)

(Subinvestigator) Taiki Kamijima, Department of Urology (ext. 2393)

(Subinvestigator) Shuhei Aoyama, Department of Urology (ext. 2393)

(Subinvestigator) Ren Toriumi, Department of Urology (ext. 2393)

(Subinvestigator) Ryunosuke Nakagawa, Department of Urology (ext. 2393)

(Subinvestigator) Masato Kizawa, Department of Urology (ext. 2393)

At night (5:30–9:00 p.m.) and on holidays, please call the emergency room (076-265-2000) and ask for the urologist on duty.

**18. Please contact the consultation service any time**

If you have any questions or concerns about this study, please do not hesitate to contact your physician or the physician responsible for this study. In addition, there are other contact points at the following locations:

Department of Urology, Tokyo Medical and Dental University Hospital

Tel: 03-5803-6111 (ext. 5295) (weekdays 8:30–17:00 except Saturdays, Sundays, and holidays)

Complaint consultation

Clinical Trial Management Center, Tokyo Medical and Dental University Hospital

Tel: 03-5803-4575 (weekdays 8:30–17:00 except Saturdays, Sundays, and holidays)

**19. Others**

The Accreditation Committee for Clinical Research is responsible for reviewing and confirming initial applications, applications for changes, applications for minor changes, periodic reports, reports of diseases, reports of major nonconformities, reports of discontinuation, reports of termination, and providing a written opinion to the principal investigator.

Name of accreditation committee: Tokyo Medical and Dental University Clinical Research Review Committee

Contact for inquiries and complaints about the accreditation committee: Clinical Trial Management Center

Tel: 03-5803-4575 (weekdays 8:30–17:00 except Saturdays, Sundays, and holidays)
