# Supplementary material for: A phase II randomized trial of metastasis-directed therapy with alpha emitter radium-223 in men with oligometastatic castration-resistant prostate cancer (MEDAL)
Source: BMC Urol. 2023 Mar 6;23:33. doi: 10.1186/s12894-023-01202-z (PMC9987040; doi:10.1186/s12894-023-01202-z)
Supplement: Supplementary file 3 — Additional file 3: The consent form given to participants. [file 12894_2023_1202_MOESM3_ESM.docx]

**Consent Form**

Title of clinical study: **“A phase II randomized trial of metastasis-directed therapy with alpha emitter radium-223 in men with oligometastatic castration-resistant prostate cancer”**

*Instructions for use*

□ Introduction: About independent clinical research (conflict of interest)

□ Purpose of this study

□ Methods of this study

□ Planned duration of participation in this study

□ Planned number of participants in this study

□ The expected effects of this treatment and the risks of adverse events that may occur.

□ Other treatment options if this treatment is not used.

□ What will happen to your health during this study (financial burden, compensation)?

□ Participation in this study is voluntary.

□ We will keep you informed about this treatment.

□ We may discontinue this treatment.

□ If you participate in this study, your medical records may be examined during or after the study.

□ Your medical records may be examined during or after the study.

□ If the results of this study are made public, your identity will not be revealed.

□ What you must do if you agree to participate in this study

□ About your cost burden

□ About your doctor

□ About the consultation service

[Patient’s signature]

To participate in this study, I have received a full explanation of the above matters, received the consent document, and fully understood the contents.

I have received sufficient explanation on the above points, received the consent document, and fully understood the contents, and I agree to participate in this study.

Patient’s name (Self-signed):

Name of substitute (signature) (relationship):

(Signature of physician)

I have fully explained this voluntary clinical research to the above patient.

Date of explanation: / / (year/month/day)

Affiliation

Name (Self-signed):

**Withdrawal of Consent Form**

Title of Clinical Research: **“A phase II randomized trial of metastasis-directed therapy with alpha emitter radium-223 in men with oligometastatic castration-resistant prostate cancer”**

I was fully informed about the “A phase II randomized trial of metastasis-directed therapy with alpha emitter radium-223 in men with oligometastatic castration-resistant prostate cancer” and agreed to cooperate in the study.

However, I would like to withdraw my consent and refuse any use of my personal data for research purposes unless there is a special reason and I give my consent after receiving an explanation.

(Patient’s signature line)

Date of withdrawal of consent: / / (year/month/day)

Patient’s name (Self-signed):

Name of substitute (signature) (relationship):

(Signature of physician)

Date of confirmation of withdrawal of consent: / / (year/month/day)

Affiliation

Name (Self-signed):
